# Inhibition of UII/UTR System Relieves Acute Inflammation of Liver through Preventing Activation of NF-κB Pathway in ALF Mice

**DOI:** 10.1371/journal.pone.0064895

**Published:** 2013-06-03

**Authors:** Dong-yu Liang, Liang-ming Liu, Chang-gen Ye, Liang Zhao, Fang-ping Yu, De-yong Gao, Ying-ying Wang, Zhi-wen Yang, Yan-yan Wang

**Affiliations:** 1 Department of Hepatology, Songjiang Hospital Affiliated to the First People’s Hospital Shanghai Jiaotong University, Shanghai, China; 2 Department of Infection, Songjiang Hospital Affiliated to the First People’s Hospital Shanghai Jiaotong University, Shanghai, China; French National Centre for Scientific Research, France

## Abstract

Urotensin II (UII) is implicated in immune inflammatory diseases through its specific high-affinity UT receptor (UTR). Enhanced expression of UII/UTR was recently demonstrated in the liver with acute liver failure (ALF). Here, we analysed the relationship between UII/UTR expression and ALF in lipopolysaccharide (LPS)/D-galactosamine (GalN)-challenged mice. Thereafter, we investigated the effects produced by the inhibition of UII/UTR system using urantide, a special antagonist of UTR, and the potential molecular mechanisms involved in ALF. Urantide was administered to mice treated with LPS/GalN. Expression of UII/UTR, releases of proinflammatory cytokines including tumor necrosis factor-alpha (TNF-α), interleukin-1 beta (IL-1β) and interferon-γ (IFN-γ), and activation of nuclear factor κB (NF-κB) signaling pathway were assessed in the lethal ALF with or without urantide pretreatment. We found that LPS/GalN-challenged mice showed high mortality and marked hepatic inflammatory infiltration and cell apoptosis as well as a significant increase of UII/UTR expression. Urantide pretreatment protected against the injury in liver following downregulation of UII/UTR expression. A close relationship between the acutely flamed hepatic injury and UII/UTR expression was observed. In addition, urantide prevented the increases of proinflammatory cytokines such as TNF-α, IL-1β and IFN-γ, and activation of NF-κB signaling pathway induced by LPS/GalN in mice. Thus, we conclude that UII/UTR system plays a role in LPS/GalN-induced ALF. Urantide has a protective effect on the acutely inflamed injury of liver in part through preventing releases of proinflammatory cytokines and activation of NF-κB pathway.

## Introduction

Acute liver failure (ALF) is a life-threatening clinical syndrome with a sudden loss of hepatic function in patients with no preexisting history of liver disease. The pathological feature of ALF is the death of large number of parenchymal hepatocytes resulting from cell apoptosis and necrosis [1]. Massive cell loss leads to functional impairment of the liver, ultimately multiorgan failure and death. Mortality is high in patients with ALF (∼90%) [2]. Currently, there are yet no special valid therapies except for emergency liver transplantation [3]. A challenge in understanding the pathophysiological mechanisms of ALF may account for the deficiency of the therapeutic methods.

As an animal model of ALF, lipopolysaccharide (LPS)/D-galactosamine (GalN)-challenged mice showed massive apoptosis in liver [4], [5]. Through crosstalking with innate immune system, the drugs can initiate the early immune injury of liver by stimulating production of proinflammatory cytokines [6]. These proinflammatory cytokines can mediate hepatic tissue inflammatory response and cell apoptosis and ultimately induce ALF in this model [7]–[9]. Thus, immune-mediated liver injury plays a pivotal role in the pathophysiology of ALF [10].

Recently, urotensin II (UII), a somatostatin-like neuropeptide, and its special UT receptor (UTR) were found to have an enhanced expression in the liver with ALF [11]. Both UII and UTR expressions are mainly found in the innate immune cells including Kupffer cells (KCs) and endothelial cells (ECs), and have a significant correlation with interferon-γ (IFN-γ) and interleukin-6 (IL-6) expression [11]. However, the role of UII/UTR system in the damage liver is not yet elucidated.

UII, initially isolated from the teleost urophysis [12], has since been identified in many classes of vertebrates, including humen [13]. UII is widely distributed within many tissues including liver [14], [15]. UII exerts biological actions under both physiological and pathological conditions. In addition to producing vasoconstriction and dilation, UII promotes fiber formation and cellular proliferation, and has an important effect on substance metabolism [16], [17]. Plasma UII is elevated in patients with hypertension [18], coronary heart disease [19], congestive cardiac failure [20], type II diabetes mellitus [21] and hepatic cirrhosis [22]. Watanabe et al [23] suggest that increased plasma UII levels are associated with pathogenesis of vascular endothelial dysfunction and tissue damage.

UII mediates its action through the specific high-affinity receptor UTR, identified as the orphan receptor G protein-coupled receptor 14 (GPR14) [24]. UTR is expressed equally and abundantly in numerous diseased conditions [16], especially in inflammatory regions of lesions [25]. Recent studies have shown an interaction between UII/UTR and the immune system. It is demonstrated that the majority of monocytes and a large portion of NK cells express UTR, being upregulated by LPS and TNF-α [25]. UII can induce chemotaxis of monocytes and monocyte-derived macrophages as a chemoattractant directly [25] or by stimulating the expressions of tissue factor (TF) and vascular cell adhesion molecule-1, (VCAM-1)/intercellular adhesion molecule-1 (ICAM-1) in ECs [26]. In addition, UII can upregulate IL-6 expression [27]. It suggests a potential immune inflammatory function of UII/UTR system.

It was recently demonstrated that the blockage of UII signal pathway protected against acute myocardial injury by using urantide [28], a special antagonist of UTR. In the present study, we investigated whether protection of the compound exists in LPS/GalN-induced ALF following inhibition of UII/UTR system in mice, and investigated whether immuno-inflammatory mechanism and NF-κB signaling pathway are involved in the protective effects.

## Materials and Methods

### Materials

LPS (*Escherichia coli* strain O55: B5) and GalN were obtained from Sigma–Aldrich (St.Louis, MO, USA). Urantide was purchased from Peptides (Louisville, KY, USA). Antibodies against UII and UTR were purchased from Santa Cruz Biotechnology (Santa Cruz, CA, USA). Antibodies against IκB-α, p-IκB-α, NF-κB p65 and β-actin were purchased from Cell Signaling Techology (Danvers, MA, USA). Male BALB/c mice (6 weeks age), with a body weight of 20 to 22 g, were obtained from the Animal Center of First People’s Hospital Affiliated to Shanghai Jiaotong University, and maintained in specific pathogen free air at a temperature of 22±2°C with 12 h light and dark cycles and relative humidity of 50%. Animals care and treatment were humanity and in compliance with the recommendations in the Guide for the Care and Use of Laboratory Animals of the National Institutes of Health. The protocol was approved by the Committee on the Ethics of Medical Scientific Research of the First People’s Hospital, Shanghai Jiaotong University (Permit Number: 2012KY041). All surgery was performed under sodium pentobarbital anesthesia, and all efforts were made to minimize suffering.

### Experimental Design

Mice were pretreated before induction of ALF intravenously with a total volume of 100 µl of normal saline (NS) or with 0.6 mg⋅kg^−1^ urantide dissolved in 100 µl NS (urantide). At 30 min after the injection, mice (NS, urantide) were challenged by an intraperitoneal injection with a total volume of 200 µl of NS (sham) or with 800 mg⋅kg^−1^ GalN and 50 µg⋅kg^−1^ LPS dissolved in 200 µl of NS as previously described [5] (LPS/GalN, urantide+LPS/GalN). Animal survival rates were calculated at 1, 2, 4, 6, 8, 12, 24 and 48 h after LPS/GalN injection, respectively. For sample, mice were anesthetized and killed at 12 h after LPS/GalN challenge, and blood and liver were collected for testing.

### Immunohistochemistry

Sections from shock-frozen tissues were stained by an indirect immunoperoxidase technique as described previously [29]. Briefly, the sections were incubated with primary antibody against UII or UTR at 4°C overnight. After washing, peroxidase-coupled secondary antibody was applied and incubated for 30 min at room temperature. Bound antibody was detected with 3, 3′-diaminobenzidine tetrachloride (DAB) (Sigma–Aldrich, St.Louis, MO, USA). All the sections were then counterstained with hemalaun. Brown-yellow staining was recognized as positive in the cells. Primary antibody was substituted by phosphate buffer saline (PBS) for negative control.

### Terminal Deoxynucleotidyl Transferase-mediated dUTP Nick-end Labeling (TUNEL) Assay

Apoptotic cells were determined by the TUNEL method using in situ apoptosis detection kit (Roche, Mannheim, Germany) according to the manufacturer’s instructions. Briefly, after fixed with 4% paraformaldehyde, liver sections were blocked by incubating with 0.03% H_2_O_2_, and permeabilized by 0.1% Triton X-100. TUNEL reaction mixture was applied at 37°C for 60 min and visualized by horse-radish peroxidase-conjugated sheep anti-fluorescein antibody (Roche, Mannheim, Germany) and DAB. The sections were then counterstained with hemalaun for 5 s. The positive cells in the stained sections were identified and counted under a light microscopy. The percentage of apoptotic cells with positive nuclei was calculated in the most frequently identified areas, and referred to as the apoptotic index (AI). At least 1000 liver cells per section were examined in five randomly selected fields by light microscopy (×400). At least four sections were used for calculating the AI means of each group.

### Caspase-3 Activity Assays

Caspase-3 activity in liver tissues was measured using Caspase Fluorometric Assay Kit (BioVision, Mountain View, CA) according to the manufacturer’s instructions. Briefly, after homogenized, liver tissue lysate was centrifuged and the supernatants were collected for determining the caspase-3 activity. 100 µg of the extracted proteins for each sample were tested in duplicate experiments with 50 µM final concentration of fluorescent substrates for caspase 3 (DEVD-AFC) at 37°C for 1∼2 h. The cleavage of substrate was monitored in a fluorescence reader using an excitation wavelength of 400 nm and an emission wavelength of 505 nm. Calibration curves were generated using standard concentrations of AFC and caspase-3 activity was calculated from the slope of the recorded relative fluorescence and expressed as relative fluorescence units (RFU).

### Reverse Transcription-polymerase Chain Reaction (RT-PCR)

Total RNA was extracted from liver tissues with TRIzol reagent (Invitrogen Carlsbad, CA, USA) following the manufacturer’s instructions. Two micrograms of total RNA were employed for synthesis of first-strand cDNA with a M-MLV RT kit (Fermentas, Canada). The PCR primers were designed by Primer Premier 6.0 software (PremierBiosoft, PaloAlto, CA, USA) from the reported sequences (GenBank™ accession number X66539 for TNF-α, NM031512 for IL-1β, NM008337 for IFN-γ, NM011910 for UII, NM145440 for UTR, NM031144 for β-actin). The primer sequences are described in table 1. PCR was performed with the following thermal cycling conditions: for UII, UTR and IL-1β, denaturation at 94°C for 5 min followed by 32 cycles of denaturation at 94°C for 1 min, primer annealing at 58°C for 45 s and primer extension at 72°C for 45 s with a final extension at 72°C for 10 min; for TNF-α and IFN-γ, denaturation at 94°C for 5 min followed by 32 cycles of 94°C 1 min, 51°C 45 s and 72°C 45 s with a final extension at 72°C for 10 min.

**Table 1:** Primer sequences used for PCR.

Genes	Primer sequences (5′ → 3′)	Productsize (bp)
UII	Sense:GAGCATTCCCTTCATCGTAG	
	Antisense : CATAGCGTTCACTGCTCATT	385
UTR	Sense: CTTTCACTCAGCACCTCAT	
	Antisense :CTTAGTTTTTCTCCACACTGTT	211
TNF-α	Sense: GGCGGTGCCTATGTCTCAG	
	Antisense: GACAAGCCTGTAGCCCACG	354
IL-1β	Sense: CTCGTGCTGTCGGACCCAT	
	Antisense: GTGGGTGTGCCGTCTTTCAT	184
IFN-γ	Sense: AGTGGCATAGATGTGGAAGA	
	Antisense: TCAAACTTGGCAATACTCAT	298
β-actin		
Primer1:	Sense: TGCCGCATCCTCTTCCTC	
	Antisense: CCACAGGATTCCATACCCAAG	249
Primer2:	Sense: CCTGGCACCCAGCACAAT	
	Antisense: GGGCCGGACTCGTCATAC	156

### Enzyme-Linked Immunosorbent Assay (ELISA)

Serum cytokine levels such as TNF-α, IFN-γ and IL-1β were quantified with an ELISA kit (R&DSystems, Abingdon, UK) according to the manufacturer’s protocol; and serum UII levels were determined using enzyme immunoassay kit (Phoenix Biotech, Beijing, China), based on the principle of a “competitive” enzyme immunoassay [30], according to the manufacturer’s guidelines.

### Nuclear and Cytoplasmic Protein Extraction

Nuclear and cytoplasmic proteins were extracted with NE-PER Nuclear and Cytoplasmic Extraction Reagents (Thermo Scientific, MA, USA) according to the manufacturer’s protocol. In brief, liver was cut into small pieces and centrifuged. The pellet was homogenized and suspended in CER I. 10 min later, ice-cold CER II was added to the tube. After vortexed twice, the tube was centrifuged. The supernatant (cytoplasmic extract) was transferred to a clean pre-chilled tube for storage. The insoluble fraction, which contained nuclei, was suspended in ice-cold NER. The tube was vortexed, incubated on ice and then centrifuged. The supernatant (nuclear extract) was transferred to a clean pre-chilled tube for storage.

### Western Blot Analysis

The cytoplasmic extract fraction was used for IκB-α and phospho(Ser32)-IκB-α protein analysis, while the nuclear fraction for NF-κB p65 protein analysis. 20 µg of proteins were subjected to SDS-PAGE and electroblotted onto PVDF membrane (Bio-Rad, Hercules, CA, USA). Immunostaining was performed using antibodies directed against IκB-α, p-IκB-α, NF-κB p65 and β-actin (Cell Signaling, Bevery, MA, USA) as the primary antibody, and a blotting detection reagent using SuperSignal® West Pico Chemiluminescent Substrate (Pierce Biotech, Rockford, USA).

### Electrophoretic Mobility Shift Assay (EMSA)

The nuclear extract fraction was used for EMSA assay according to the manufacturer’s protocol and previous report [31] with minor modifications. Binding reactions were performed by incubating the nuclear extracts in reaction buffer (1× binding buffer, 2.5% Glycerol, 5 mM MgCl_2_, 50 ng/µl Poly(dI·dC), 0.05% NP-40) with the biotin-labeled DNA probe (20 fmol) for 20 min at room temperature. The products were electrophoresed on a 4.8% polyacrylamide gel in 0.5× TBE. Binding reactions in the gel were electrophoreticly transferred to Nylon membrane. The membrane was crosslinked at 120 mJ/cm^2^ using a UV-light crosslinking instrument equipped with 254 nm bulbs. Biotin-labeled DNA was detected by chemiluninescence. An NF-κB consensus oligonucleotide (5′-AGTTGAGGGGACTTTCCCAGGC-3′) from the mouse IgGκ-light chain was purchased and labeled (Shenggong Biotech, Shanghai, China).

### Statistical Analysis

SPSS17.0 statistical software was used in the study. The results are expressed as means ± standard deviation (SD). A *P* value less than 0.05 was considered statistically significant.

## Results

### UTR Inhibitor, Urantide, Prevents LPS/GalN-induced Liver Injury

**Figure 1:**
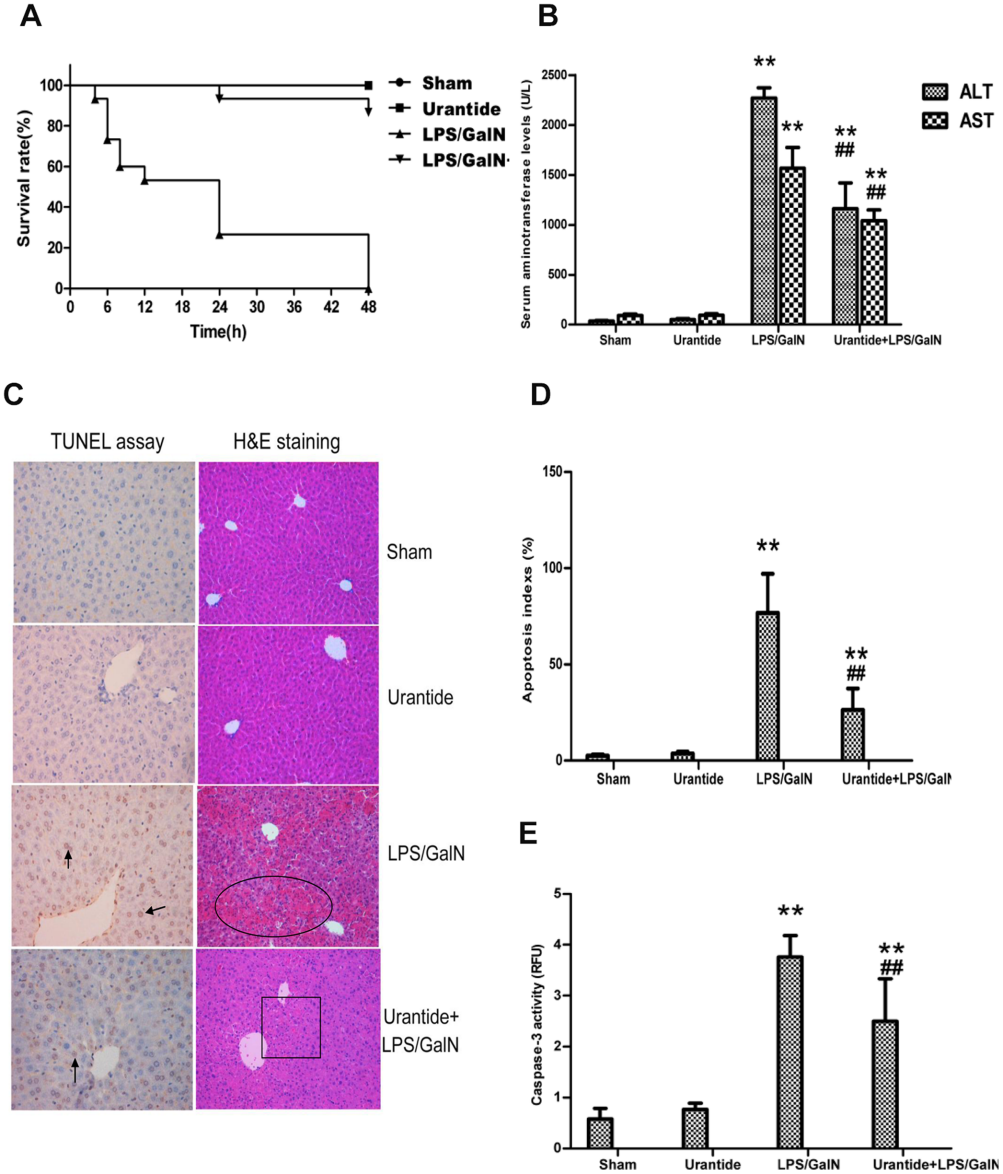
Effects of urantide on survival, hepatic injury and apoptosis. The mice were treated with urantide or vehicle 0.5 h before LPS/GalN injection. (A) Survival; mouse survival rates in four groups at different times within 48 h after challenged with LPS/GalN (n = 15 each group). (B) Serum levels of ALT and AST; Data represent means ± SD (n = 6 each group). (C) Morphological appearance; Hematoxylin and Eosin (H&E) staining of the liver (right): circle area indicates hemorrhagic necrosis, and pane area shows inflammatory infiltration change (magnification 200×). TUNEL assay of liver (left): arrowhead indicates apoptotic hepatocytes (magnification 200×). (D) Apoptotic indexes from liver TUNEL assay; bars represent means ± SD (n = 6 each group). (E) Caspase-3 activity of liver; Bars represent means ± SD (n = 6 each group). *P<0.05 and **P<0.01 versus sham; ^#^ *P*<0.05 and ^##^ *P*<0.01 versus LPS/GalN.

Administration of LPS and GalN caused 100% lethality within 48 h after combinative injection, whereas the urantide pretreatment definitely protected against the mortality in mice injected with LPS/GalN. 87 percent of urantide-pretreated mice (13/15) survived at 48 h after the LPS/GalN challenge (Fig. 1A). LPS/GalN injection increased serum levels of alanine aminotransferase; (ALT) and aspartate aminotransferase. (AST). The pretreatment of urantide significantly reduced the releases of ALT and AST into the circulation (Fig. 1B). The injection of LPS/GalN induced marked hepatic injuries accompanied by haemorrhage, massive necrosis and inflammatory infiltration. Pretreatment with urantide prevented these pathological changes (Fig. 1C). In the mice treated with urantide alone, no obvious morphological manifestation was displayed in the liver.

We also examined hepatocyte apoptosis in the mouse livers via TUNEL combined with caspase-3 activity. The results indicated that the injection of LPS/GalN induced massive hepatocyte apoptosis and increased the apoptotic index and caspase-3 activity of the liver; whereas urantide pretreatment markedly reduced these effects (Fig. 1D and E).

### Expression of UII/UTR System is Inhibited by Urantide Pretreatment in LPS/GalN-changed Mice

**Figure 2:**
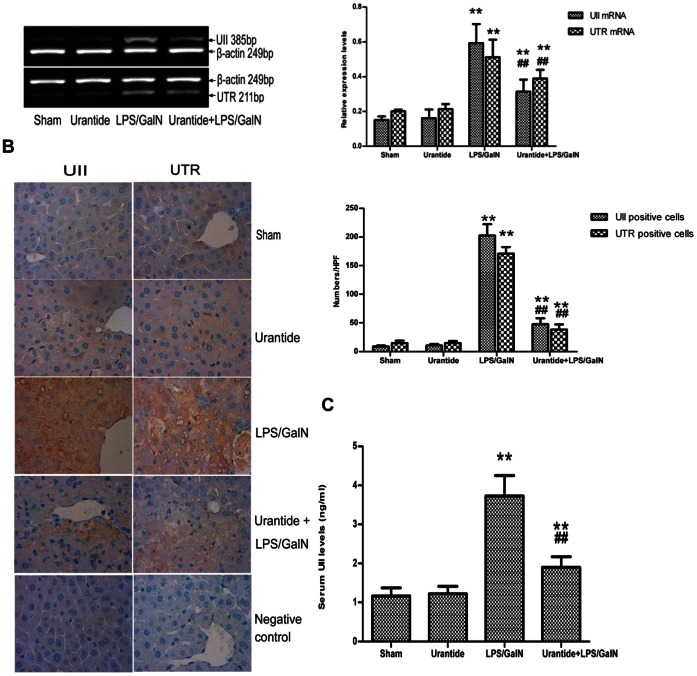
Effects of urantide on UII/UTR system expression. (A) mRNA expression of UII and UTR in liver; left panel shows a representative ethidium bromide-stained gel of RT-PCR products, and right shows relative expression levels of UII and UTR mRNA in liver after normalization to β-actin. Data represent means ± SD (n = 6 each group). (B) Immunohistochemistry of UII (left) and UTR (right) in liver; The positive cells are stained with yallow-brown color (magnification ×400). Quantification of positive signals in UII or UTR staining is shown in the bottom panel. Data represent means ± SD (n = 6 each group). (C) Levels of UII secretion in blood. The mouse serum were assayed for UII secretion via ELISA. Data represent six independent studies. Values are mean ± SD (n = 6). *P<0.05 and **P<0.01 versus sham; ^#^ *P*<0.05 and ^##^ *P*<0.01 versus LPS/GalN. HPF, high power field.

Both UII and UTR were only faintly expressed in livers of sham and urantide mice. After challenged by LPS/GalN, a strong expression of UII/UTR was found in the mouse livers (Fig. 2A, B). UII/UTR expression included endothelial cells of arteries, veins and bile ducts, as well as sinusoidal lining cells (Fig. 2B). No expression of UII/UTR was found on hepatocytes and lymphocytic infiltrates. Predominant staining included cell membrane and cytoplasm. The prior administration of urantide significantly inhibited the increase of liver UII/UTR expression. Hypersecretory serum UII was also inhibited by urantide pretreated in LPS/GalN-challenged mice (Fig. 2C).

### Production of Proinflammatory Cytokines is Inhibited by Urantide Pretreatment in LPS/GalN-challenged Mice

**Figure 3:**
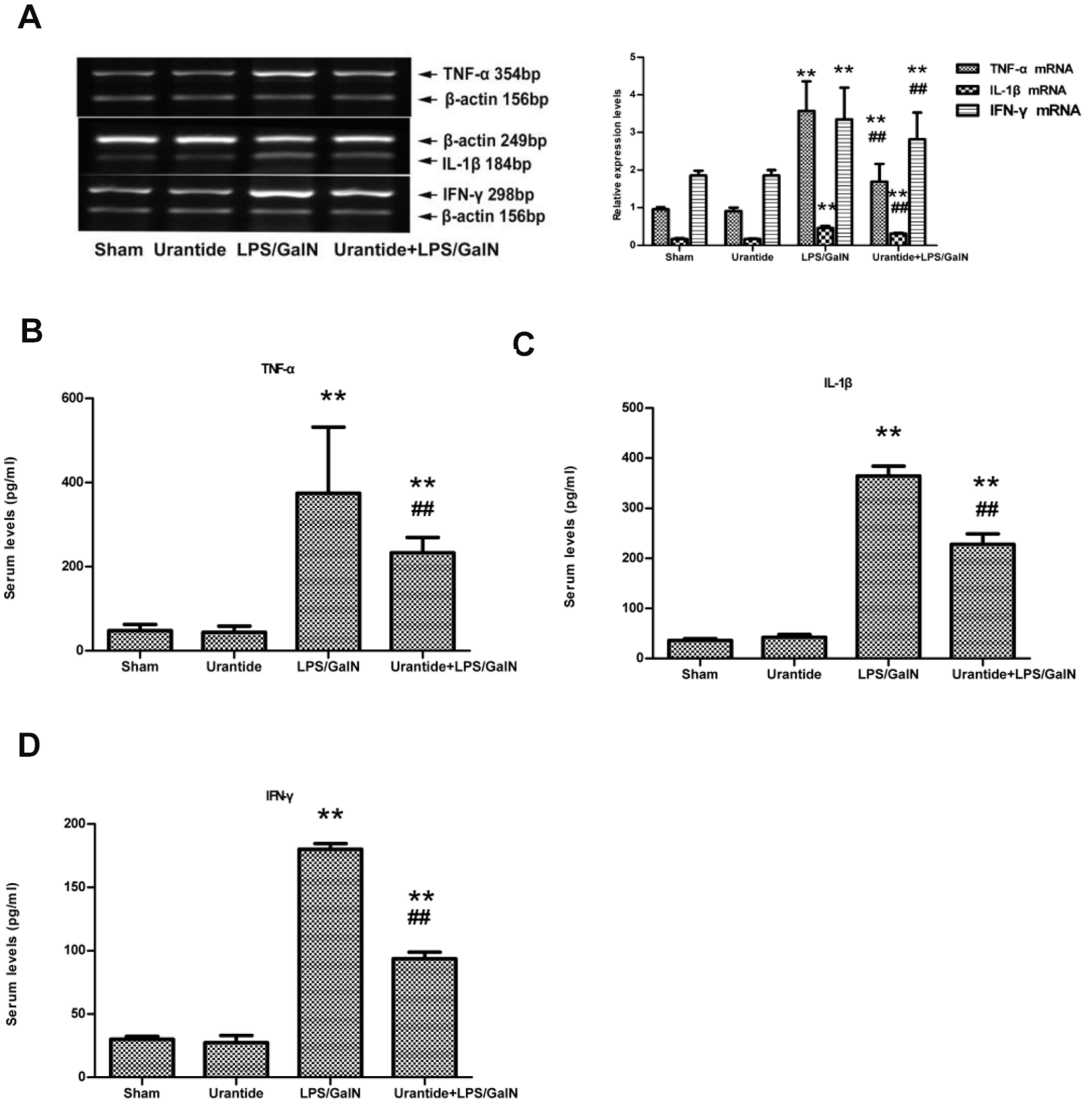
Effects of urantide on levels of pro-inflammatory cytokines in liver and serum. (A) TNF-α, IL-1β and IFN-γ mRNA expression in liver; Left panel shows a representative ethidium bromide-stained gel of RT-PCR products. Relative expression levels of these cytokines in liver are shown in the right panel after normalization to β-actin. (B) Serum levels of TNF-α; (C) Serum levels of IL-1β; (D) Serum levels of IFN-γ. Bars represent means ± SD (n = 6). *P<0.05 and **P<0.01 versus sham; ^#^ *P*<0.05 and ^##^ *P*<0.01 versus LPS/GalN.

Liver expressions of TNF-α, IL-1β and IFN-γ mRNA increased after LPS/GalN treatment. Urantide pretreatment inhibited the overexpressions of these cytokines (Fig. 3A). We also analyzed the serum levels of TNF-α, IL-1β and IFN-γ. Serum concentrations of TNF-α, IL-1β and IFN-γ significantly increased after LPS/GalN treatment. Urantide pretreatment prevented the increases of these cytokines (Fig. 3B, C and D).

### Activation of Liver Nuclear Factor-κB Pathway is Inhibited by Urantide Pretreatment in LPS/GalN-challenged Mice

**Figure 4:**
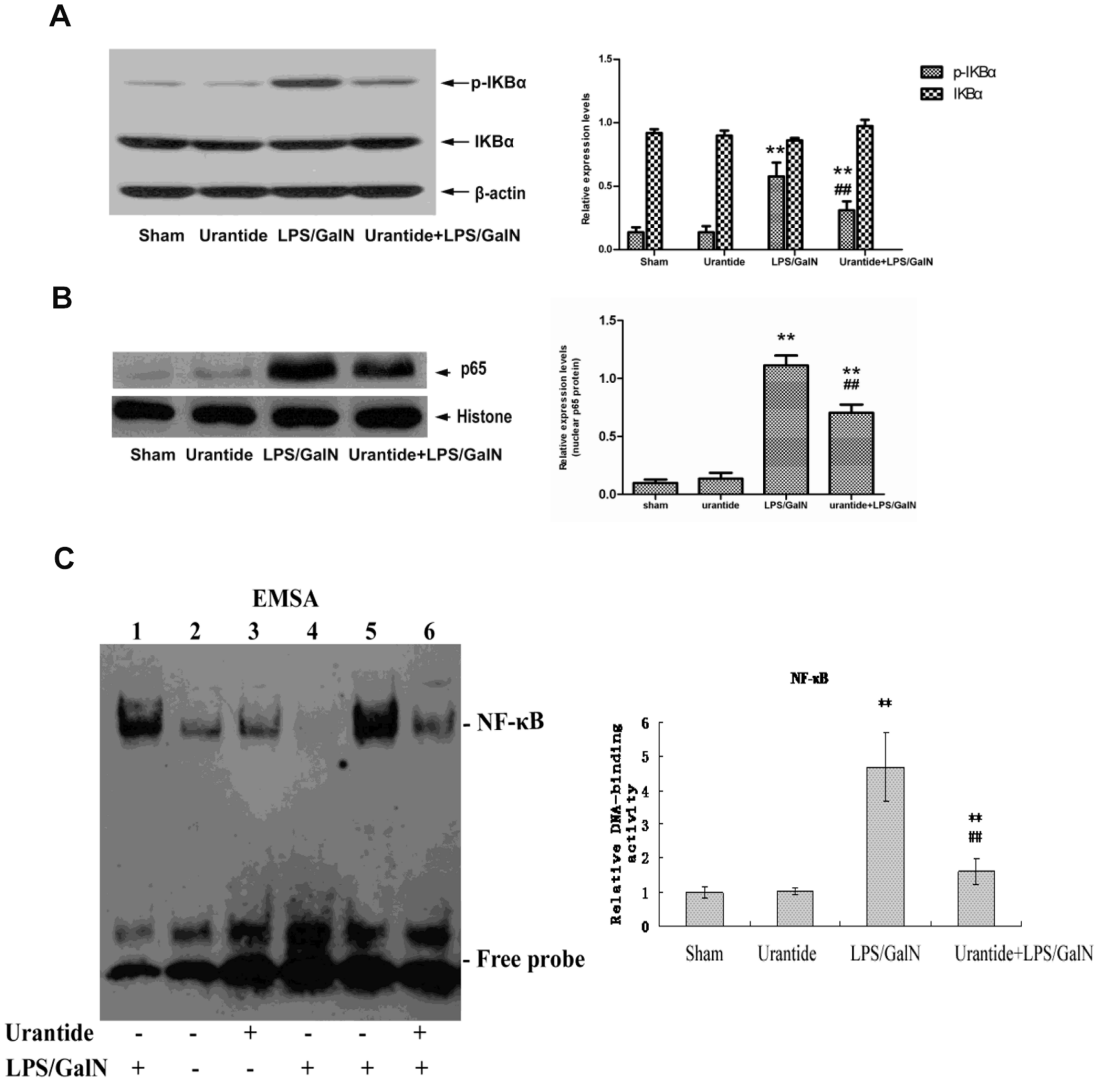
Effect of urantide on NF-κB pathway activation in liver. (A) IκB and phospho- IκB in liver cytoplasmic protein extracts; Left panel shows a representative picture of Western blot, and right shows the relative levels of IκBα and phospho- IκBα protein in liver after normalization to β-actin. (B) NF-κB p65 subunit in liver nuclear protein extracts; Left panel shows a representative picture of Western blot, and right shows the relative levels of p65 protein in liver after normalization to histone. (C) DNA-binding activity of NF-κB in liver nuclear protein extracts; DNA-binding activity of NF-κB was analyzed by EMSA (top). Lane 2, 3, 5 and 6 are target reactions (liver nuclear extract+biotin-DNA probe). Lane 2: sham; lane 3: urantide; lane 5: LPS/GalN; lane 6: urantide+LPS/GalN. Lane 1 and 4 are control reactions. Lane 1: cold competitive reaction of mutation DNA probe (liver nuclear extract+biotin-DNA +200-fold molar excess of unlabeled mutation DNA); lane 4: cold competitive reaction of DNA probe (liver nuclear extract+biotin-DNA +200-fold molar excess of unlabeled DNA). The bands corresponding to NF-κB were quantitated by densitometry (lower). Bars represent means ± SD (n = 6). *P<0.05 and **P<0.01 versus sham; ^#^ *P*<0.05 and ^##^ *P*<0.01 versus LPS/GalN.

To investigate the underlying mechanisms by which urantide exerts its inhibitory effects on the induction of cytokines in liver injury, we examined the effect of urantide on NF-κB pathway activation in the liver. LPS/GalN stimulated the phosphorylation of IκBα, upstream inhibitive protein of NF-κB, and urantide decreased the levels of phosphorylation (Fig. 4A). Nuclear translocation of NF-κB p65 subunit was stimulated by GalN/LPS. Urantide treatment inhibited the increases of nuclear levels of the protein (Fig. 4B). EMSA with liver nuclear extracts revealed that urantide inhibited the DNA-binding activation of NF-κB stimulated by LPS/GalN treatment (Fig. 4C).

## Discussion

UII is recently shown to play a role in the immune mechanisms of tissue injury [32], [33]. In ALF, marked upregulation of UII and its receptor UTR have been demonstrated in patients [11]. The role of UII/UTR system, however, has never been evaluated in ALF. Our approach using urantide pretreatment has allowed us to investigate the role of this system in LPS/GalN-challenged mice.

In this experiment, we found that co-administration of LPS/GalN induced a marked inflammation and liver injury as well as upregulation of UII and UTR. Urantide pretreatment not only protected against the LPS/GalN-induced hepatic injury by inhibiting inflammatory infiltration of liver and hepatocyte apoptosis, but also resulted in downregulation of UII/UTR. Therefore, UII/UTR system may have a key role in the acute inflamed liver. It has been demonstrated that both UII and UTR are expressed in the same type cells including KCs and ECs [11], [34], the important compoment of innate immunity system. A autocrine/paracrine stimulatory effect may exit in these UII/UTR-expressing cells during ALF. The importance of hepatic KCs has been highlighted for initiating and driving liver inflammatory response by releasing proinflammatory cytokines [35]. Among the cytokines that can induce inflammation, TNF-α plays a privileged role in the acute injury of liver [8] and EC activation [35]. EC activation or dysfunction can further lead to releases of proinflammatory cytokines and other inflammatory mediators, and devastate the immune inflammatory injury of liver [36].

However, the effect of UII/UTR system on proinflammatory cytokines is not fully understood. In the study, we found marked increases of TNF-α, IL-1β and IFN-γ in the peripheral circulation and liver after LPS/GalN challenge. UII/UTR system inhibition using urantide significantly reduced the levels of these proinflammatory cytokines. Therefore, the releases of these cytokines may be a consequence of UII/UTR system activation induced by LPS/GalN. Moreover, IFN-γ can mediate UII/UTR system upregulation [37]. From the hepatic cytokine milieu induced by LPS/GalN challenge, a positive feedback loop may exit between LPS/GalN-induced activation of UII signal pathway and the proinflammatory cytokine expression, resulting in prolonged massive inflammatory injury in the liver and a vicious cycle. The protection of urantide pretreatment may result from an interruption of the vicious cycle via blocking UII signal transduction in LPS/GalN-induced ALF.

To determine the mechanisms underlying the protective effect of UII/UTR system inhibition in LPS/GalN liver injury, we examined the effect of urantide on the signaling molecules of NF-κB pathway in liver. NF-κB transcription factor has been considered as a central mediator of inflammatory process and a key participant in innate and adaptive immune responses [38], [39]. Under the unstimulated circumstances, NF-κB is sequestered in the cytoplasm through interaction with the special inhibitor IκBα; within the liver, in response to LPS stimulation, KCs are activated through special receptor TLR4 to induce phosphorylation and degradation of IκBα [40], and the inactive NF-κB-IκBα complex is dissociated, therefore allowing free NF-κB component RelA (p65) to translocate to the nucleus [41], [42]. Once activated, NF-κB binds to target DNA sequences and induces transcriptional expression of various inflammatory cytokines, such as TNF-α, IL-1β, IL-6 and IFN-γ [43], [44], resulting in hepatic acute inflammatory injury. In our study, the inhibition of UII/UTR system using urantide suppressed IκBα phosphorylation, p65 nuclear translocation and NF-κB DNA-binding activity induced by LPS/GalN in the liver, revealing that urantide pretreatment reduces the expressions of cytokines and protects against hepatic inflammatory injury mainly through the inhibition of NF-κB activation.

Thus, we conclude that UII/UTR system may play a pivotal part in the pathogenesis of LPS/GalN-induced ALF. UTR antagonist, urantide, has protective and anti-inflammatory effects on the lethal liver injury through preventing releases of proinflammatory cytokines including TNF-α, IL-1β and IFN-γ due to inhibition of NF-κB activation.
